# KinesioTaping after botulinum toxin type A for cervical dystonia in adult patients

**DOI:** 10.1002/brb3.2541

**Published:** 2022-03-03

**Authors:** Małgorzata Dec‐Ćwiek, Karolina Porębska, Katarzyna Sawczyńska, Marcin Kubala, Magdalena Witkowska, Kinga Zmijewska, Jakub Antczak, Joanna Pera

**Affiliations:** ^1^ Department of Neurology Medical College Jagiellonian University Krakow Poland; ^2^ Faculty of Medicine and Health Sciences Andrzej Frycz Modrzewski Krakow University Krakow Poland; ^3^ Department of Orthopedics and Physiotherapy Medical College Jagiellonian University Krakow Poland

**Keywords:** botulinum injection, botulinum toxin, cervical dystonia, kinesiotaping, neurorehabilitation

## Abstract

**Introduction:**

Studies explored physiotherapeutic approaches in cervical dystonia (CD) patients with or without botulinum toxin (BoNT) injections, however the results are varying. There are no clinical trials investigating the effects of kinesiology taping in CD patients. The objective of this study is to investigate the efficacy of kinesiology taping as an adjunct to the BoNT injections in patients with CD.

**Methods:**

Twenty‐five patients were enrolled to the study. Patients were randomly assigned to the experimental 1 (BoNT + KinesioTaping), experimental 2 (BoNT + ShamTaping) or control (BoNT) treatment. After 12 weeks they were moved to the next experimental group and finally every patient received all 3 proposed treatment options. The severity of CD was quantified with the *Toronto Western Spasmodic Torticollis Rating Scale* (TWSTRS) including Torticollis severity, Disability, and Pain scales. Quality of life was evaluated using *Craniocervical dystonia questionnaire* (CDQ4).

**Results:**

In all treatment groups, there was a significant improvement in dystonia symptoms measured with TWSTRS (total score) after BoNT injection regardless of the allocation to the experimental treatment (*p* < .05). ANOVA analysis revealed no differences in any of the TWSTRS variables after the intervention. Quality of life was significantly improved after application of taping (*p* < .05, *p* = .03).

**Conclusions:**

Application of KinesioTaping after BoNT injection provided no additional effect on the severity of dystonia, although the quality of life was improved in patients with CD. Further research investigating the effect of KinesioTaping prior to BoNT injection is required.

## Graphical abstract

There are no clinical trials investigating the effects of kinesiology taping in CD patients. The objective of this study is to investigate the outcome of kinesiology taping in addition to BoNT injections in patients with CD. Application of KinesioTaping after BoNT injection provided no effect on the severity of dystonia and quality of life in patients with CD.

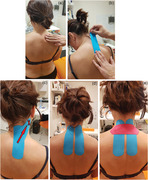

## 1 INTRODUCTION

Cervical dystonia (CD), the most common form of adult‐onset focal dystonia, is a movement disorder characterized by involuntary contractions of the cervical muscles due to a dysfunction of sensorimotor neural circuits. It causes twisting and repetitive movements of the neck and head and may be accompanied by tremor. Sometimes, CD results in abnormal postures (Albanese et al., 2015). Apart from motor symptoms, 36% of patients experience marked nonmotor symptoms such as psychiatric features (anxiety, depression, behavioral and cognitive problems), pain, sexual dysfunction or sleep impairment (Klingelhoefer et al., 2014). Seventy to ninety percent of patients develop the symptoms of CD between the age of 40 and 60 years. Females are twice more affected than males (Chan et al., 1991).

Motor and nonmotor symptoms of CD significantly impair daily functioning and cause embarrassment frequently leading to social withdrawal. Recent studies have shown a negative impact of CD on patients’ quality of life (Ben‐Shlomo et al., 2002; Muller et al., 2002; Pekmezovic et al., 2009; Van Den Dool et al., 2016).

CD treatment options offer inadequate effectiveness with scarce patient satisfaction. Chemodenervation with botulinum neurotoxin injection (BoNT) is a worldwide accepted standard of care for patients with CD. BoNT exerts its therapeutic effects by blocking neuromuscular acetylcholine transmission at the peripheral nerve terminals. The therapeutic response becomes apparent within 1–2 weeks after the BoNT injection, with peak effects at approximately 4–6 weeks and a gradual decline in outcome thereafter (Albanese et al., 2011; Greene et al., 1990; Poewe et al., 1998; Poewe et al., 2016; Simpson et al., 2016). BoNT product guidelines currently recommend at least 12 weeks intervals between injections (http://www.ipsen.com; http://allergan‐web‐cdn‐prod.azureedge.net). Thus, patients with CD treated with BoNT experience a rollercoaster effect, as they receive treatment with waning effectiveness over time that increases again following the subsequent injection (Francisco et al., 2021). Clearly, BoNT treatment meets a limited patient satisfaction. It seems meaningful to administer an adjunctive therapy that would maintain a near steady‐state level of treatment outcome. For example, to enhance the effects of BoNT, physical therapy may be considered as a supplementary treatment (Crowner et al., 2007; Jankovic et al., 2006; Ressman et al., 2000; Smania et al., 2003; Tassorelli et al., 2006). Available studies explored rehabilitative approaches in CD patients with or without BoNT injections; however, the results are varying (Boyce et al., 2013; Counsell et al., 2016; De Pauw et al., 2014; Hu et al., 2019; Tassorelli et al., 2006).

Kinesiology taping, known as an alternative taping technique, involves a combination of tension applied along the tape and stretching of the target muscle. That, amongst others, results in a change of recruitment activity patterns of the muscles and alleviates prolonged muscle contraction and even postural deviation (Kase et al., 2016).

Kinesiology taping is currently used in rehabilitation of patients suffering from different neurological diseases. For instance, combining BoNT to the spastic equinovarus foot with kinesiotaping results in better outcome than applying sham taping (Karadag‐Saygi et al., 2010; Kase et al., 2016). Low BoNT doses followed by ankle‐foot taping is as effective as the injection of higher BoNT doses for the foot inversion with positive effects on gait parameters (Reiter et al., 1998).

To the best of our knowledge, there are no clinical trials investigating the effects of kinesiology taping on motor symptoms in CD patients. The objective of this study is to investigate the efficacy of kinesiology taping as an adjunct to the BoNT injections in patients with CD.

## 2 MATERIAL AND METHODS

### 2.1 Participants

The study was designed as a single‐centre, prospective, evaluator‐blind, randomized, crossover trial. Ethical approval was granted by the institutional review board.

The participants were recruited from the Movement Disorders outpatient clinic of the Department of Neurology, Collegium Medicum, Jagiellonian University in Krakow between January 2019 and January 2021. Participants provided a written, informed consent. Demographic characteristics, medical history including age at disease onset and course of disease, genetic factors, duration of the treatment with BoNT injection were recorded during the initial visit during which a neurological examination was performed. Exclusion criteria included unfinished diagnostic process, presence of segmental, multifocal, generalized dystonia, or hemidystonia, history of receiving deep brain stimulation treatment or neck surgeries, the presence of contraindications for kinesiology taping (wounds, fresh scars, allergies to acrylic glue, tape intolerance).

### 2.2 Study design

Patients were randomly assigned to three groups with different therapeutic schemes: experimental 1 (BoNT + KinesioTaping), experimental 2 (BoNT + ShamTaping) or control (BoNT + no taping) treatment. After 12 weeks patients were moved to the next group and eventually every patient received all 3 proposed treatment options.

**FIGURE 1:**
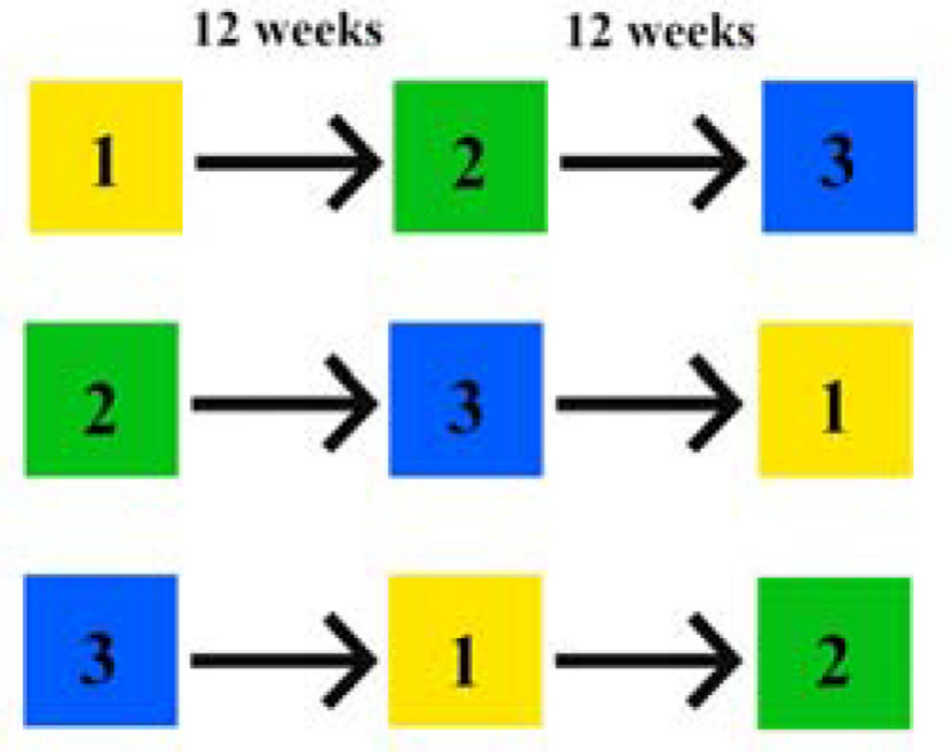
The study design. Experimental groups: 1, experimental treatment 1 (BoNT+KinesioTaping); 2, experimental treatment 2 (BoNT + ShamTaping); 3, control (BoNT + no taping)

The randomization sequence was created using a computer‐generated random number, with 1:1:1 allocation of individuals to either intervention groups or the control group. Subjects were assessed 2 times per cycle: at the BoNT injection visit and after 6 weeks. The randomization process and study design are summarized in Figure 1.

Outcome assessors and patients were unaware of treatment type, whilst the physiotherapist was informed on group assignments.

### 2.3 Procedures

Injections were performed every three months with the use of USG guidance. Muscles for BoNT injections were chosen individually accordingly to collum‐caput (*Col‐Cap*) concept subtype of CD; the scheme of BoNT injections and doses of BoNT were constant throughout the study (when subjects switched the experimental group, the scheme of injection and BoNT doses remained unchanged).

**FIGURE 2:**
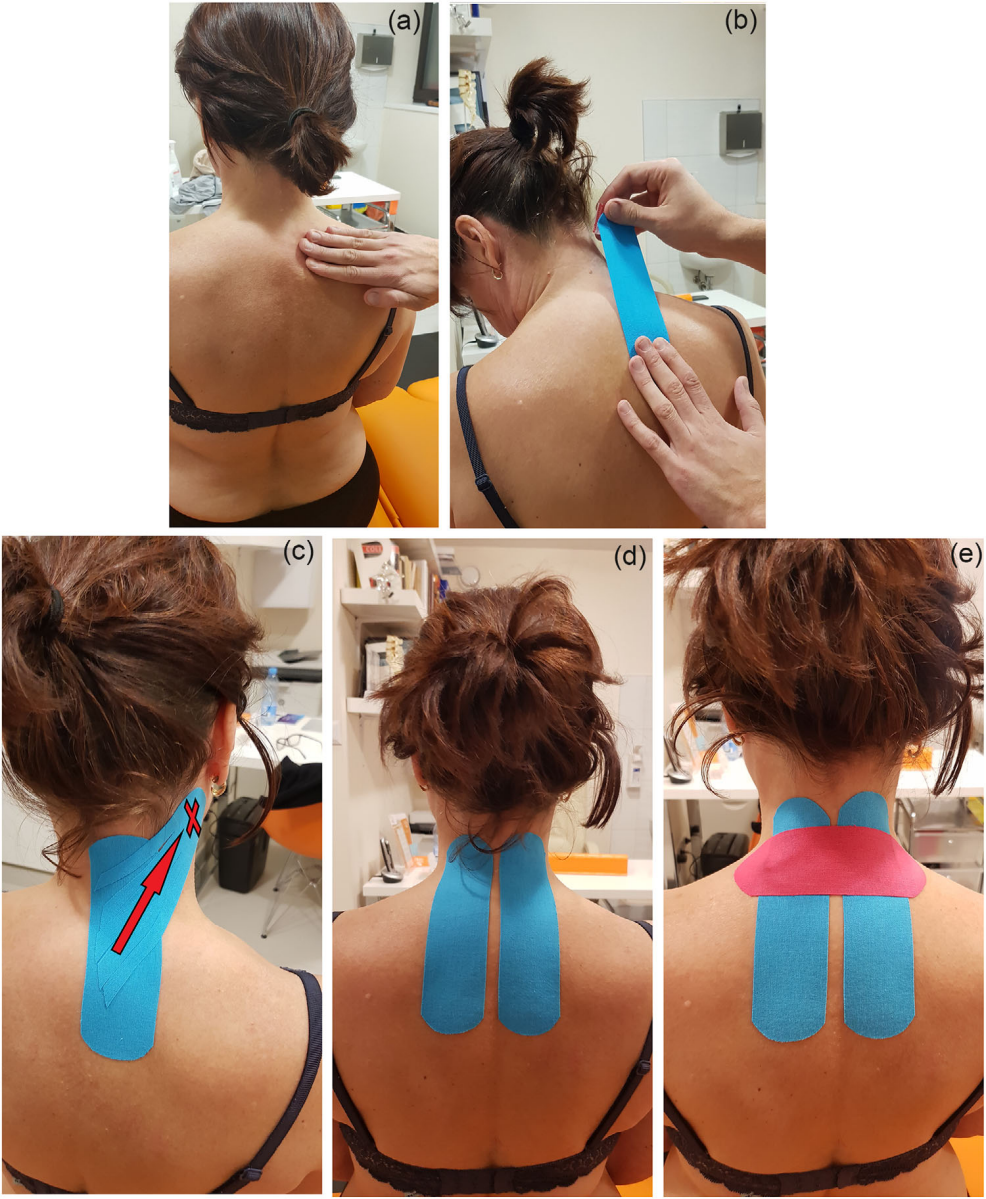
KinesioTaping (a–c). A patient with laterocaput on the right and torticaput on the left. (a) The assessment of fascial sliding over the semispinalis cervicis muscle. (b) The application of tape using the muscle technique. (c) Right splenius capitis muscle taped. The arrow shows direction of the tape impact on the fascia and the muscle. The tape application was made from deep to superficial muscles. The x mark indicates the base of the tape. ShamTaping (d and e). Application of two vertical (d) and one horizontal (e) slices without any tension and without moving the head or neck

Kinesiology taping was performed seven days after BoNT injection and for four consecutive weeks once per week by the same, experienced physiotherapist (see Figure 2). In the experimental group 1, patients were treated with the kinesiology tape using the dynamic taping methods according to an established schedule. The application of kinesiology taping was performed using the muscle technique on individual muscles or muscle groups acting synergistically. Taping was applied in the direction of fascial restriction (to the restriction or from the restriction) according to the subjective assessment of the patient (reduction of involuntary movements within the head and neck, improvement of the posture of the C‐Th segment of the spine and shoulder girdle). The physiotherapist slid a fascia over a given muscle or muscle group and assessed the patient's symptoms, then the base of tape was glued so that the tail of tape pulls to the base according to the therapeutic fascia slide. All patients in this group were also taped using the analgesic technique (ligament technique) in the area of the C‐Th spine or the area of the shoulder complex, which was subjectively indicated as the most painful area of the body. The technique was executed with a single transverse application or double cross application (45°–90°), which means applying the central part of the tape with a tension of 75–100% while the two ends are glued without tension. If the patient did not report subjective pain symptoms, the application was omitted. In the experimental group 2, patients were taped, but in a nontherapeutic manner. The tape application was done without tension and without moving the head or neck, including stretching the muscles in the form of two vertical slices and one horizontal slice glued to the C‐Th area of the spine.

The severity of the CD was quantified with the *Toronto Western Spasmodic Torticollis Rating Scale* (TWSTRS), including Torticollis severity scale (score range, 0–35), Disability scale (score range, 0–30), Pain scale (score range, 0–20). TWSTRS motor severity was video recorded for blinded rating by two independent movement disorder neurologists.

Quality of life was assessed with 24‐item *Craniocervical dystonia questionnaire* (CDQ4), that covers five domains: stigma, emotional wellbeing, pain, activities of daily living, and social/family life. Each item consists of five statements representing increasing severity of impairment, scored from 0 to 4.

Statistical analysis was carried out with the use of PS Imago Pro 6.0 statistical package. Categorical data were presented as counts and percentages. Continuous data were presented as mean and standard deviation. Due to limited sample size a nonparametric Kruskal–Wallis test was used for comparisons. Differences were considered to be statistically significant if the two‐sided *p* value was less than .05.

## 3 RESULTS

Twenty‐five patients diagnosed with primary CD were initially enrolled to the study, albeit there were six dropouts. The reasons for exclusion were: inability to attend taping caused by logistic complications (*n* = 2), unfinished second cycle of taping due to COVID‐19 restrictions (*n* = 3), taping‐related skin rash (*n* = 1). As a result, data from 19 patients were analyzed. The demographic and clinical features of patients are summarized in Table 1.

**TABLE 1:** Patients’ demographics and clinical characteristics

**Total number of patients**	19
**Sex (M/F)**	4/15
**Age (mean ± SD in years)**	54.7 ± 12.4
**Disease duration in years (mean ± SD)**	27.8 ± 12.4
**No. of BoNT injections (mean, range)**	19 (range: 6–56)
**Comorbidities (no. of patients)**:	
Depression	3
Cervical spodylosis	3
Arterial hypertension	2
Others (Graves’ disease, high cholesterol, atherosclerosis, nephrolithiasis)	6
**Subtype of CD**	**Number of patients (%)**
Torticaput	14 (73.7%)
Laterocaput	11 (57.9%)
Anterocollis	6 (31.2%)
Retrocaput	6 (31.2%)
Laterocollis	5 (26.3%)
Anterocaput	5 (26.3%)
Torticollis	4 (21%)
Retrocollis	1 (5.3%)
Sagital shift	5 (26.3%)
**Other features**	
Tremor “no‐no”	7 (36.8%)
Tremor “yes‐yes”	2 (10.5%)
Shoulder elevation	6 (31.2%)

All but two patients presented more than one subtype pattern of cervical dystonia, two subtypes—five patients (26,3%), three subtypes—eight patients (42,1%), and four subtypes—four patients (21%), respectively.

## 4 TWSTRS

In all treatment groups, there was a significant improvement in dystonia symptoms as measured with TWSTRS (total score) after BoNT injection regardless of the allocation to the experimental treatment (BoNT + KinesioTaping, BoNT + ShamTaping, control; *p* < .05). According to the subscales of TWSTRS, there was a marked difference only in the Torticollis severity scale (*p* < .05) in all experimental treatment groups. There was no statistically relevant difference on the Disability (*p* = .55, *p* = .23, and *p* = .07, respectively) or Pain scale (*p* = .32, *p* = .22, and *p* = .22, respectively).

ANOVA analysis revealed no differences in any of TWSTRS variables after the intervention (see Table 2).

**TABLE 2:** Comparison of TWSTRS scores between experimental groups

	1	2	3	*p*
**Baseline**				
Torticollis severity scale score	5.76 (± 3.60)	6.47 (± 4.39)	5.11 (± 3.90)	.575
Disability scale score	4.88 (± 3.46)	7.32 (± 5.12)	5.53 (± 4.43)	.344
Pain scale score	5.41 (± 4.00)	5.32 (± 3.54)	5.39 (± 3.10)	.996
Total TWSTRS score	16.06 (± 9.01)	19.11 (± 9.76)	15.97 (± 8.79)	.500
**After intervention**				
Torticollis severity scale score	−12.47 (± 4.13)	−12.95 (± 4.59)	−12.79 (± 3.74)	.941
Disability scale score	−1.88 (± 5.01)	0.11 (± 2.49)	−0.68 (± 1.57)	.300
Pain scale score	−1.04 (± 3.07)	−0.67 (± 3.41)	−0.67 (± 2.30)	.911
Total TWSTRS score	−15.40 (± 8.51)	−14.36 (± 6.87)	−14.17 (± 4.21)	.842

1, experimental treatment 1 (BoNT + KinesioTaping); 2, experimental treatment 2 (BoNT + ShamTaping); 3, control (BoNT + no taping) < .005.

**FIGURE 3:**
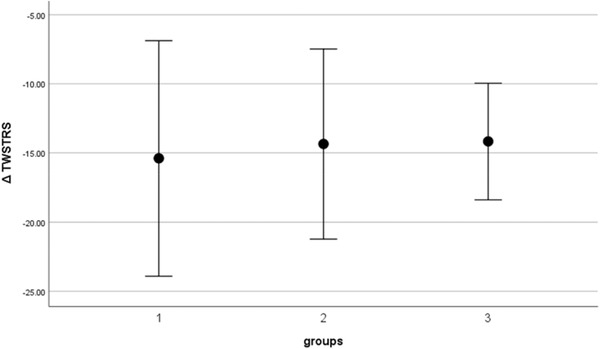
Comparison of Δ TWSTRS score calculated as a difference between TWSTRS score at the BoNT injection visit and TWSTRS score at the follow‐up visit in three experimental groups. 1, experimental treatment 1 (BoNT + KinesioTaping); 2, experimental treatment 2 (BoNT + ShamTaping); 3, control (BoNT + no taping)

Figure 3 illustrates the changes in TWSTRS score calculated as a difference between TWSTRS score at the BoNT injection visit and TWSTRS score at the follow‐up visit in the three experimental groups.

### 4.1 CDQ24

Quality of life was significantly improved from baseline after application of taping (*p* < .05 and *p* = .03). Considering individual domains of CDQ24, KinesioTaping improved “stigma” and “emotional wellbeing” after the intervention in experimental treatment group 1 (both *p* < .05). Whereas, in the ShamTaping group, “stigma” and “activities of daily living” domains were markedly bettered (both *p* = .02; Table 3).

**TABLE 3:** Comparison of the individual domain scores and of the total CDQ24 score between experimental groups

	1	2	3
**Baseline (absolute values)**			
Stigma domain	5.71 (± 4.81)	6.32 (± 4.81)	5.79 (± 4.88)
Emotional wellbeing domain	3.82 (± 3.01)	4.37 (± 3.40)	3.89 (± 2.87)
Pain domain	1.82 (± 2.24)	2.16 (± 2.17)	1.79 (± 1.87)
Activity of daily living domain	4.65 (± 3.92)	5.89 (± 4.16)	4.47 (± 3.08)
Social/family domain	1.88 (± 1.97)	2.16 (± 3.04)	1.37 (± 1.86)
Total CDQ‐24 score	17.88 (± 13.34)	20.89 (± 14.31)	17.32 (± 11.42)
**After intervention (change from baseline)**			
Stigma domain	−2.41 (± 3.74)	−2.68 (± 3.71)	−1.68 (± 4.44)
Emotional well‐being domain	−1.25 (± 2.56)	−2.05 (± 3.06)	−0.47 (± 2.46)
Pain domain	−0.76 (± 1.72)	−0.32 (± 2.95)	−0.42 (± 1.92)
Activity of daily living domain	−2.12 (± 3.55)	−0.32 (± 2.31)	−0.95 (± 2.44)
Social/family domain	0.18 (± 0.88)	−0.37 (± 1.71)	−0.84 (± 2.01)
Total CDQ‐24 score	−6.35 (± 9.43)	−5.74 (± 8.37)	−4.42 (± 10.15)

1, experimental treatment 1 (BoNT + KinesioTaping); 2, experimental treatment 2 (BoNT + ShamTaping); 3, control (BoNT + no taping).

No side effects were observed following taping (except for one subject who experienced a skin rash after the first tape application), and patients reported positive feedback on treatment acceptability.

## 5 DISCUSSION

In this single‐center, prospective, evaluator‐blind, randomized, crossover study on the effect of KinesioTaping in patients with CD, we did not observe superior efficacy of taping as an adjunctive therapy to BoNT injection versus BoNT alone. Although no improvement was seen in the objective outcome measures, patients’ quality of life evaluated using CDQ24, a patient‐reported outcome, was ameliorated. In other words, patients perceived a subjective improvement after treatment, however without outcome improvement when taping was applied. Dystonic movements in CD are caused by cocontraction of muscles that can be classified into three groups depending on their type of involvement: dystonic, antagonist, and compensatory. Muscles identified as responsible for pathological posture should be stretched and relaxed by the treatment procedure comprising BoNT injection and physiotherapy (Bleton et al., 2010; Tatu et al., 2007). KinesioTaping, which relies on applying tension along the tape and placing the target muscle in a stretched position, is considered a physiotherapeutic method. Despite its wide clinical use, there is little evidence of the efficacy of KinesioTaping in CD. Indeed, there are only two studies exploring the effect of KinesioTaping in patients with focal cervical neurological disorder (Giray et al., 2017; Pelosin et al., 2013). Giray et al. investigated the outcome of KinesioTaping in addition to therapeutic exercises for the treatment of congenital muscular torticollis, a rare musculoskeletal disorder characterized by unilateral shortening of the sternocleidomastoid muscle. Infants were randomized to one of three groups, which followed a different rehabilitation programme (therapeutic exercises, therapeutic exercises with KinesioTaping applied only to the affected side, and therapeutic exercises + KinesioTaping applied to the affected and unaffected sides). In the end, no group demonstrated superiority over others for all outcome measures. Authors concluded that KinesioTaping did not add any beneficial effect to exercise therapy in terms of muscle function of lateral flexors of the neck in infants. Although the findings of that and the current research are congruent, it is difficult to compare the results due to different pathophysiology of the diseases and studied populations (Giray et al., 2017). Pelosin et al. evaluated the effectiveness of KinesioTaping on nonmotor functions in 25 patients with focal dystonia not treated with BoNT injections. The patients were randomized to a 14‐day treatment with KinesioTaping or ShamTaping over affected muscles (neck muscles in CD patients or forearm muscles in focal hand dystonia patients), and after a 30‐day washout period, received other treatment. Compared to ShamTaping, KinesioTaping decreased the subjective sensation of pain and modified the ability of sensory discrimination (Pelosin et al., 2013).

Abnormal sensorimotor cortical plasticity contributes to the pathophysiology of dystonia (Edwards et al., 2006; Quartarone et al., 2003). Kojovic et al. (2011) reported that BoNT injections into neck muscles decreased sensorimotor associative plasticity in the hand area in patients with CD. This central effect was mediated by changes in motor maps caused by reduced afferent input from neck muscles after the injections.

The blockade of neuromuscular acetylcholine transmission at the nerve terminals following BoNT injections results in a marked reduction of afferent input. This, in turn, stimulates reprogramming of central circuity from a peripheral approach. Taping, instead, is thought to stimulate the cutaneous mechanoreceptors (Halseth et al., 2004). Such an activation causes local depolarization that triggers signal transmission along the afferent fibers traveling towards the central nervous system to the sensorimotor area. One can speculate that the effect induced by BoNT on the sensorimotor area is primary. However, this key effect is not augmented by adjunctive KinesioTaping, although the signal transmission provoked by mechanoreceptors activation is sent towards the same sensorimotor area as afferent feedback produced by BoNT. Therefore, we did not observe superior effect of combined BoNT and KinesioTaping compared to BoNT treatment alone as the effect of BoNT injections is not enhanced by tape application at the central level.

On the other hand, according to the KinesioTaping method manual, skin traction caused by the tape promotes an elevation of the epidermis (Hu et al., 2019). The pressure on the mechanoreceptors, located below the dermis, is reduced so that the stimulation of the receptors and afferent nerve transmission decreases. A change in abnormal sensorimotor plasticity at the cortical level induced by reduced afferent projection may be associated with clinical improvement. When afferent projection is inhibited after BoNT injection, it would be worth investigating if KinesioTaping before the BoNT procedure gives rise to a synergistic effect in patients with CD.

Disability in CD, that affects function, activities, participation, environmental and personal factors, undoubtedly influences patients’ quality of life. A recent study that analyzed patient's perspective on BoNT injection treatment in CD revealed that symptom re‐emergence has a significant impact on daily activity and quality of life. Responders indicated that they would prefer a regimen with longer injection intervals highlighting the unmet need for long lasting symptom relief in CD (Comella et al., 2021). Although our study failed to demonstrate an objective improvement with the use of KinesioTaping in patients with CD treated with BoNT, we observed a markedly improved quality of life, a patient‐reported outcome measured by CDQ24, in patients who underwent combined BoNT injection and KinesioTaping, absent when patients were treated BoNT injection only. Such a result may be explained by the placebo effect of taping as CDQ24 score was improved in all patients in whom taping was administered regardless of whether it was correct or sham application. Investigating the impact of treatment of BoNT injection and KinesioTaping on inter‐injection intervals is warranted.

We acknowledge that this study had limitations such as a small sample size and the use of TWSTRS for rating dystonia severity. Accordingly, this research was designed to be evaluator‐blind, randomized and crossover to overcome the weakness of a small number of patients participating in the study.

TWSTRS does not enable the evaluation of dystonic tremor that was present in some patients. What is more, TWSTRS does not weigh dystonic pattern according to the *Col‐Cap* concept. The *Col‐Cal* concept, established on the basis of CT/MRI imaging examination and functional anatomy, identifies eight major subtypes of CD (Finsterer et al., 2015; Reichel, 2011).

In summary, application of KinesioTaping after BoNT injection provided no effect on the severity of dystonia although it subjectively improved quality of life measures in patients with CD. We suspect that blockade on afferent nerve transmission induced by BoNT was responsible for reduced effect of KinesioTaping. Further research on a larger group of patients also investigating the effect of KinesioTaping prior to BoNT injection is required.

## CONFLICT OF INTEREST

The authors declare no potential conflicts of interest with respect to the research, authorship, and/or publication of this article.

## FUNDING

The authors received grant from CMUJ for the research and /or publication of this article (N41/DBS/000200).

The authors confirm that data supporting the findings of this study are available within the article.
